# Complement Factor H Family Proteins Modulate Monocyte and Neutrophil Granulocyte Functions

**DOI:** 10.3389/fimmu.2021.660852

**Published:** 2021-10-04

**Authors:** Éva Kárpáti, Mariann Kremlitzka, Noémi Sándor, Dávid Hajnal, Andrea E. Schneider, Mihály Józsi

**Affiliations:** ^1^ Department of Immunology, ELTE Eötvös Loránd University, Budapest, Hungary; ^2^ MTA-ELTE Complement Research Group, Eötvös Loránd Research Network (ELKH), Department of Immunology, ELTE Eötvös Loránd University, Budapest, Hungary

**Keywords:** complement, cytokine, neutrophil extracellular trap (NET), factor H (FH), factor H-related protein (FHR), monocyte, neutrophil granulocyte, extracellular DNA

## Abstract

Besides being a key effector arm of innate immunity, a plethora of non-canonical functions of complement has recently been emerging. Factor H (FH), the main regulator of the alternative pathway of complement activation, has been reported to bind to various immune cells and regulate their functions, beyond its role in modulating complement activation. In this study we investigated the effect of FH, its alternative splice product FH-like protein 1 (FHL-1), the FH-related (FHR) proteins FHR-1 and FHR-5, and the recently developed artificial complement inhibitor mini-FH, on two key innate immune cells, monocytes and neutrophilic granulocytes. We found that, similar to FH, the other factor H family proteins FHL-1, FHR-1 and FHR-5, as well as the recombinant mini-FH, are able to bind to both monocytes and neutrophils. As a functional outcome, immobilized FH and FHR-1 inhibited PMA-induced NET formation, but increased the adherence and IL-8 production of neutrophils. FHL-1 increased only the adherence of the cells, while FHR-5 was ineffective in altering these functions. The adherence of monocytes was increased on FH, recombinant mini-FH and FHL-1 covered surfaces and, except for FHL-1, the same molecules also enhanced secretion of the inflammatory cytokines IL-1β and TNFα. When monocytes were stimulated with LPS in the presence of immobilized FH family proteins, FH, FHL-1 and mini-FH enhanced whereas FHR-1 and FHR-5 decreased the secretion of TNFα; FHL-1 and mini-FH also enhanced IL-10 release compared to the effect of LPS alone. Our results reveal heterogeneous effects of FH and FH family members on monocytes and neutrophils, altering key features involved in pathogen killing, and also demonstrate that FH-based complement inhibitors, such as mini-FH, may have effects beyond their function of inhibiting complement activation. Thus, our data provide new insight into the non-canonical functions of FH, FHL-1, FHR-1 and FHR-5 that might be exploited during protection against infections and in vaccine development.

## Introduction

The complement system is a fundamental part of innate immunity, the main function of which is to discriminate self and non-self structures in order to keep immune homeostasis. Recognition of foreign particles and modified host cells by complement results in its activation *via* the classical, lectin or alternative pathways, leading to the cleavage of the central component C3 into C3a and C3b. C3 is the major effector of the complement cascade, causing inflammation by releasing the anaphylatoxin C3a or clearance of the target structures by opsonization with C3b and allowing for further propagation of the cascade, potentially leading to target cell lysis (1). Although complement activation is necessary to eliminate altered self and foreign structures, its over-stimulation might result in attack on the host itself and cause tissue injury. Hence a strict control of complement activation is necessary to maintain immune balance and preserve host integrity (2).

Factor H (FH) is the major regulator of the alternative pathway (AP) of complement by acting as a cofactor for Factor I mediated cleavage of C3b and accelerating the decay of AP C3 and C5 convertases either in solution or when bound to host surfaces (3). FH consists of 20 short consensus repeats (SCR): the N-terminal SCR1-4 domains bind to C3b and exert complement regulatory activity, while the C-terminal SCR19-20 domains ensure binding to host surfaces *via* recognizing highly sialylated molecules (4, 5). In addition, FH can recognize other polyanionic molecules, like proteoglycans, glycosaminoglycans and other components of the extracellular matrix (6–9), extending the FH mediated protection also to tissues and extracellular spaces. Moreover, to exploit the potential of FH as a therapeutic agent, an artificial mini-FH was generated that contains the regulatory (SCR1-4) and ligand binding (SCR19-20) domains of FH (10, 11). Besides acting as a complement regulator, FH also exerts non-canonical functions *via* binding to various immune cells, and modulating their activation and function (12). Among those are monocytes and neutrophils, two key components of innate immunity which, similar to complement proteins, circulate in the blood to detect potentially dangerous structures and regulate inflammatory processes (13).

Binding of FH to neutrophils has been reported to modulate a wide range of their functions, among them phagocytosis, ROS production and release of antimicrobial peptides, thus FH may directly influence the ability of the cells to kill pathogens (14–16). FH exerts the vast majority of these effects in immobilized form, serving as an adhesion ligand for the cells, however in soluble form, it is mainly ineffective. Indeed, the increased adherence observed on FH covered surfaces induces rapid polarization of neutrophils, elevation in intracellular Ca^2+^ levels followed by rapid spreading on FH-coated surfaces (14, 16). Similarly, immobilized FH was shown to enhance the release of IL-8 (16), secretion of the antimicrobial peptide lactoferrin and release of hydrogen-peroxide (14), as well as the uptake of *Streptococcus pneumoniae* (17). Additionally, FH itself also promotes neutrophil migration (16). Besides phagocytosis and intracellular killing, neutrophils can trap and eliminate microorganisms by releasing neutrophil extracellular traps (NETs) (18–20). NETs induce microbial killing by histones and granule-derived antimicrobial proteins, such as myeloperoxidase (MPO), neutrophil elastase and matrix metalloproteinases that associate with these chromatin fibers (20–23). In contrast to the enhanced activation and cytokine secretion, interestingly, the release of NETs by both phorbol-myristate acetate (PMA) or fibronectin plus β-glucan-stimulated neutrophils is decreased in the presence of immobilized FH (16). Similarly to neutrophils, FH was shown to influence a wide plethora of monocyte and macrophage functions too, such as chemotaxis (24), stimulation of respiratory burst (25) and cytokine secretion (26), mainly *via* the complement receptors CR3 (CD11b/CD18) and CR4 (CD11c/CD18) (14, 27). Later, FH binding to *Mycobacterium bovis* and *Candida albicans* has been shown to play a role in the evasion of the innate immune system and in parallel, to enhance the pro-inflammatory (IL-1β, IL-6, TNFα) cytokine response of macrophages (27, 28). Monocytes can also produce extracellular traps (MoETs), mainly to enhance killing of extracellular pathogens (29–31). It was shown that FH could bind to NET and MoET directly (29, 32) and bound FH decreases the secretion of the inflammatory cytokine IL-1β in monocytes (29). These data suggest that FH can exert both pro- and anti-inflammatory activities on monocytes and neutrophils, allowing fine-tuning of cellular functions for the regulation of inflammation.

FH belongs to a protein family that also includes the FH splice variant FH-like protein 1 (FHL-1) and five FH-related proteins (FHRs), FHR-1 to FHR-5 (33–35). Similar to FH, they are also exclusively composed of SCRs. Although the complement regulatory region SCR1-4 of FH is not conserved in the FHRs, their C-terminal domains show various degree of sequence identity with C-terminal SCRs of FH. This region represents the major cell surface binding- and C3b recognition part of FH, suggesting that the main function of FHRs is to compete with FH for surface binding and thus modulate complement activation (33, 36). Indeed, both FHR-1 and FHR-5 are competitive inhibitors of FH on different ligands such as C3b, C-reactive protein, pentraxin 3, DNA and extracellular matrix components (37–41). The FH/FHR encoding gene cluster is prone to genetic rearrangements, which are associated with distinct human disorders such as atypical hemolytic uremic syndrome, C3 glomerulopathy, age-related macular degeneration and other autoimmune/inflammatory diseases (33, 42, 43). Due to their role in various diseases, the interest in the FHR molecules has increased, both on the genetic and functional level. Despite the described role of FH in modulating neutrophil and monocyte responses, only a few data are available how FHRs influence the function of these cells. For example, FHR-1 was shown to enhance the adhesion of neutrophils to *Candida albicans*, causing increased lactoferrin and ROS generation (15), suggesting an FHR-1 mediated regulation of inflammation. Recently, FHR-1 was shown to activate the NLRP3 inflammasome in monocytes when bound to necrotic cells (44).

Considering that both the FHRs and neutrophils and monocytes are key players of inflammatory diseases, we aimed to investigate whether similar to FH, the related proteins FHL-1, FHR-1 and FHR-5, as well as the recently developed mini-FH, a potential complement therapeutic, are able to modulate neutrophil and monocyte functions.

## Materials and Methods

### Proteins, Abs, Dyes and Reagents

Mini-FH was cloned into the pBSV-8His baculovirus expression vector (45), and produced in *Spodoptera frugiperda* (Sf9) cells and purified by nickel-affinity chromatography as described previously (10). FHL-1 protein, which was expressed recombinantly in *Pichia pastoris* and purified according to the methodology described previously (46, 47), was kindly provided by Dr. Christoph Schmidt (Ulm University, Germany). Recombinant human FHR-1 and FHR-5 were obtained from Novoprotein (Summit, NJ). Bovine serum albumin (BSA) was from Applichem (Darmstadt, Germany), and human serum albumin (HSA) and fibronectin (FN) were from Sigma-Aldrich (Budapest, Hungary), respectively. Purified human FH, iC3b, polyclonal goat anti-human FH antibody and polyclonal anti-histone H4 (citrulline 3) were purchased from Merck-Millipore (Burlington, MA). Mouse anti-human FH antibodies were purchased from Enzo (C18; Lausen, Switzerland) and Quidel (A254; San Diego, CA), respectively. Goat anti-human FHR-5 antibody was from R&D Systems (McKinley Place, MN). Alexa-488-conjugated rabbit anti-goat IgG, Alexa-488-conjugated donkey anti-mouse IgG and Alexa-647-conjugated goat anti-rabbit IgG were from Molecular Probes-Invitrogen (Carlsbad, CA). Phorbol-myristate acetate (PMA) and lipopolysaccharide (LPS) were from Sigma-Aldrich. Sytox Orange and Phalloidin Alexa-488 were from Thermo Scientific (Waltham, MA).

### Cells

Human neutrophil granulocytes were isolated from whole blood and human monocytes from buffy coats of healthy blood donors according to standard procedures (16, 48). Samples were provided by the Hungarian National Blood Transfusion Service after acquiring written informed consent from all donors. The study was conducted in accordance with the respective national authority (TUKEB ETT, permission number 838/PI/12). Cell purity was investigated by flow cytometry using anti-CD14 and anti-CD16 antibodies (BD Biosciences, Germany) followed by the corresponding secondary antibodies, and was higher than 90% in each case.

The U937 human monocytic cells and U937 cells overexpressing CR3 (CR3+ U937) (49) were kindly provided by Drs. Karita Haapasalo (University of Helsinki, Finland) and Carla J. C. De Haas (University Medical Center Utrecht, the Netherlands). These cell lines were maintained in RPMI 1640 medium (Lonza, Basel, Switzerland) supplemented with 10% FBS (Euroclone) under humidified air with 5% CO_2_ at 37°C. Cell viability was assessed regularly by trypane blue dye exclusion.

### Flow Cytometry

To detect FHL-1, FHR-1, FHR-5 and mini-FH binding to neutrophil and monocyte cell surfaces, 5×10^5^ monocytes and 1×10^6^ neutrophils were first incubated with 300 nM of each protein for 20 minutes at 20°C in Ca^2+^ and Mg^2+^ containing DPBS (Lonza). After washing the cells with DPBS, Fc receptor blocking reagent (Miltenyi Biotec, Germany) was added to reduce the amount of nonspecific Ab binding. Bound proteins were detected *via* incubation with the polyclonal goat anti-human FH Ab in DPBS containing fetal bovine serum (FBS, 0.1%, EuroClone, Pero, Italy) for 15 minutes at 4°C, washed and labelled with Alexa-488-conjugated rabbit anti-goat antibody (Thermo Scientific, Waltham, MA) under the same conditions. Samples were measured on FACSCalibur flow cytometer (BD Biosciences, Mountain View, US) and analyzed by FCSExpress software, version 7 (BD Biosciences).

To detect the competition between FH and FHR-1 or FHR-5, we used Alexa-488 conjugated FH generated by the Alexa Fluor™ 488 Antibody Labeling Kit according to the manufacturer’s instructions (Invitrogen). First, 5×10^5^ neutrophils were incubated with FHR-1 or FHR-5 at increasing concentrations between 300 and 5,000 nM in Ca^2+^ and Mg^2+^ containing DPBS (Lonza) for 20 min at 4°C, followed by, without washing, 300 nM Alexa-488 conjugated FH for an additional 20 min at 4°C. After washing, bound FH was measured on FACSCalibur flow cytometer. The geomean of the FH sample without competitor was set as 100%.

To compare the expression level of CR3 between the U937 and U937 CR3+ cell lines, APC-conjugated mouse anti-human CD11b (Pharmingen) antibody was added to 5×10^5^ cells at 5 µg/ml concentration for 30 min at 4°C. Fc-receptor blocking reagent (Miltenyi Biotec, Germany) was added to reduce the amount of nonspecific Ab binding.

To detect the binding of FH, FHR-1 and FHR-5 to the U937 cell lines, 5×10^5^ cells were first incubated with 50 µg/ml FH or FHR-1, or 10 µg/ml FHR-5, for 30 min at 20°C in Ca^2+^ and Mg^2+^ containing modified Hanks’s buffer, as described previously (16). After washing, Fc-receptor blocking reagent (Miltenyi) was added to reduce the amount of nonspecific Ab binding. Bound FH and FHR-1 proteins were detected by incubation with 1:250 diluted polyclonal goat anti-human FH Ab (Merck) for 30 min at 4°C, followed by 1:1000 diluted Alexa488-conjugated rabbit anti-goat Ig (Thermo Scientific) also for 30 min at 4°C. Bound FHR-5 was detected using goat anti-human FHR-5 antibody (R&D Systems) followed by the Alexa488-conjugated rabbit anti-goat Ig under the same conditions.

To detect the presence of C3-fragments on cell surfaces, 5×10^5^ U937 and CR3+ U937 cells were left untreated or incubated with 20 µg/ml iC3b (30 min on ice). After washing, the cells were incubated with polyclonal anti-C3 (Cappel, West Chester, PA) or anti-C3c (Dako, Santa Clara, CA) antibodies, both diluted 1:100, for 30 minutes at 4°C, then washed and incubated with Alexa488-conjugated rabbit anti-goat Ig or Alexa-488-conjugated donkey anti-rabbit Ig (Invitrogen) secondary antibodies, both diluted 1:500, under the same conditions. Samples were measured on Cytoflex flow cytometer (Beckman Coulter) and analyzed by FlowJo_V10 software.

### Visualization of NET Formation

To visualize the effect of FH family members on PMA-induced NET formation, 300 nM of each protein (FHR-1, FHR-5, FHL-1, mini-FH or, as negative control, HSA) was immobilized on borosilicate chambered cover glass microplates (NUNC, Rochester, NY) overnight at 4°C. NET formation was induced by addition of 100 nM PMA in solution for 3 hours and labelled with Sytox Orange following previously described protocols (16). To confirm NET formation and exclude the potential detection of nuclear DNA, antibodies against histones [a well-accepted marker of NET formation (21)] were used. After Sytox Orange staining, samples were fixed with 4% PFA for 10 minutes, washed twice and blocked with PBS containing 5% BSA and Fc receptor blocking reagent (Miltenyi Biotec) for 30 minutes at 37°C. Thereafter citrullinated H4 histones were stained with rabbit polyclonal anti-H4 antibody (citrulline 3; 1:500 dilution in DPBS) for 1 h at 20°C and with the appropriate Alexa-488 conjugated goat anti-rabbit Ig secondary Ab (1:1000) for 30 minutes at 20°C in dark. After extensive washing, NETs were investigated with an Olympus FluoView 500 laser-scanning confocal microscope (Hamburg, Germany) equipped with argon ion laser (488nm) and two He-Ne lasers (with 543 and 632 nm excitation wavelengths, respectively). Fluorescence and DIC images were acquired using a 20× objective. Images were processed by ImageJ (http://rsbweb.nih.gov/ij) and Photoshop softwares.

### Quantification of NET Production

To quantify NET production, neutrophils were incubated on FH/FHR-coated 96-well black plate (Greiner Bio-One, Kremsmünster, Austria) in the presence or absence of 100 nM PMA for 3 hours. After incubation, each well was treated with 1 U/ml Micrococcal Nuclease (Thermo Scientific) for 10 minutes at 37°C to release NETs attached to the cell surface. The reaction was stopped by applying 5 mM EDTA. After digestion, cells were pelleted and separated from the released NET by centrifugation at 1800 *g* for 8 minutes. The cell-free NET was then labeled with 5 mM Sytox Orange for 20 minutes at 37°C. Fluorescence was measured on a Fluoroscan Ascent FL microplate reader (Thermo Scientific) with excitation and emission filters of 543 nm and 592 nm, respectively. Maximal NET release was obtained after 100 nM PMA treatment, set as 100%. In case of each sample, relative fluorescence % was calculated compared to the 100% signal of PMA treatment.

### Measurement of Neutrophil Spreading

Borosilicate chambered coverglass microplates were coated with 300 nM of each protein (FHR-1, FHR-5, FHL-1, mini-FH or, as negative control, BSA) in Ca^2+^ and Mg^2+^ containing DPBS (Lonza) at 4°C overnight. Neutrophil spreading was monitored by phalloidin Alexa-488 staining with an Olympus FluoView 500 laser-scanning confocal microscope as described earlier (16). The phalloidin staining was carried out according to the manufacturer’s instructions. The contact zone areas were quantified from 150 cells in each experiment using ImageJ software with Analyze Particle tool.

### Measurement of Cytokine Production

96-well cell culture plates (Greiner Bio-One) were coated with 300 nM of FHR-1, FHR-5, FHL-1, mini-FH or, as negative control, with FN in Ca^2+^ and Mg^2+^ containing DPBS (Lonza) at 4°C, overnight. After extensive washing, neutrophils or monocytes (2×10^5^ cells/well) were added in 200 µl of RPMI 1640 (Invitrogen) supplemented with 10% FBS and gentamycin (Lonza) and cultured for 24 hours at 37°C in a humidified atmosphere containing 5% CO_2_. As a positive control, cells were stimulated with 10 ng/ml LPS. To measure the combined effect of immobilized proteins (FHR-1, FHR-5, FHL-1 and mini-FH) and soluble LPS on cytokine production, LPS was added for 24 hours after starting culturing the cells on FH/FHRs coated surfaces. Cytokine production was measured after 24 hours of stimulation, using commercial sandwich ELISA kits (R&D Systems). ELISAs were carried out according to the manufacturer’s instructions.

### Statistical Analysis

Statistical analysis was performed using GraphPad Prism version 5.00 for Windows (GraphPad Software, San Diego, California). A *p* value < 0.05 was considered statistically significant. The specific statistical tests applied are indicated in the Figure legends.

## Results

### FH Family Members Bind to Human Neutrophils and Monocytes

**Figure 1:**
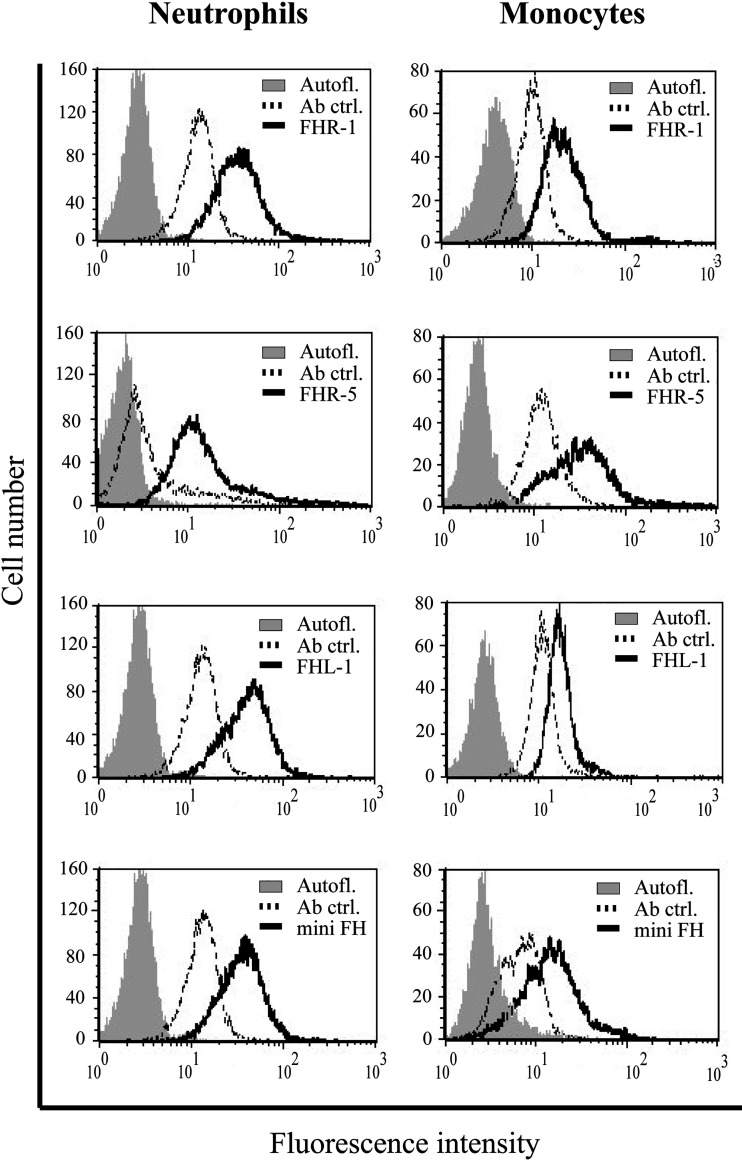
FHR-1, FHR-5, FHL-1 and mini-FH bind to primary neutrophil granulocytes and monocytes. Binding of 300 nM of each protein (solid lines) to neutrophil granulocytes and monocytes in Ca^2+^ and Mg^2+^ containing DPBS was measured by flow cytometry. Filled histograms indicate autofluorescence, dotted lines show samples incubated without the proteins added (antibody controls). Binding of the added proteins (solid black line) was analyzed using polyclonal anti-FH Ab for FHR-1, FHL-1 and mini-FH detection, and monoclonal anti-FHR-5 for FHR-5 detection. A representative histogram out of three independent experiments is shown in each case.

The binding of FH to various immune cells, especially to monocytes and neutrophils, is known for long (14–17, 24, 25, 27, 28, 50–52). However, the binding of the FHR proteins to primary cells has only been poorly studied (15, 53, 54). To investigate whether other FH family members are able to bind to neutrophils and monocytes similar to FH, freshly isolated cells were incubated with the recombinant proteins and their binding was detected by flow cytometry. FHR-1, FHR-5, FHL-1 and mini-FH, as well as FH used as a positive control, bound to both monocytes and neutrophils with high efficiency (**Figure 1**). Many factors are likely to affect the binding of these investigated FH family members *in vivo*, thus different baseline signals were observed in donors, but binding of the recombinant FHRs could always be detected.

### The Role of CR3 in Binding FH Family Proteins

**Figure 2:**
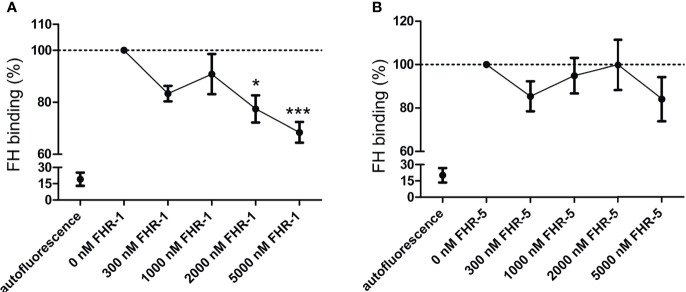
Competition between FHR-1/FHR-5 and FH for binding to neutrophils. Neutrophils were preincubated with increasing concentrations of **(A)** unlabeled recombinant FHR-1 or **(B)** FHR-5, then fluorescence-labelled FH (50 µg/ml; 300 nM) was added to the cells. Bound FH was detected by flow cytometry. Data represent means ± SEM from five **(A)** and four **(B)** independent experiments. Differences with *p* < 0.05 were considered statistically significant and compared to the FH treated samples without competitor protein (one-way ANOVA with Dunnett’s multiple comparison test, *p < 0.05***p < 0.001).

Previously, CR3 was described to be a receptor for both FH and FHR-1 on human neutrophils based on inhibition of binding by CR3-specific mAbs (15), and also for FH on macrophages using siRNA (27). To study whether FHR-1 or FHR-5 can inhibit FH binding to the cells, human neutrophils were incubated with fluorescence-labelled FH in the absence or presence of increasing concentrations of recombinant FHR-1 and FHR-5. At higher FHR-1 concentrations a significant decrease of FH binding to neutrophils was detected, indicating at least partially overlapping binding sites of FH and FHR-1 on the cells, whereas FHR-5 was not able to inhibit FH binding (**Figure 2**).

**Figure 3:**
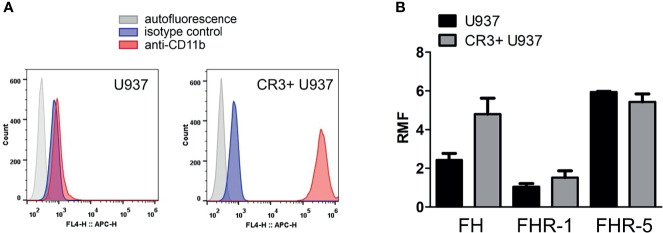
Binding of FH, FHR-1 and FHR-5 to U937 and CR3+ U937 cells. **(A)** The expression of CR3 on U937 human monocytic cells and U937 cells overexpressing CR3 (CR3+ U937) was analyzed by flow cytometry using an anti-CD11b antibody. Representative histograms are shown. **(B)** Binding of purified FH (50 µg/ml) and recombinant FHR-1 (50 µg/ml) and FHR-5 (10 µg/ml) to U937 cells and CR3+ U937 cells was measured by flow cytometry. RMF, relative mean fluorescence. Data are means + SEM from three independent experiments.

Since FH family proteins all bind to various C3-fragments, and such C3-fragments might be present on the cells isolated from human blood, we wanted to exclude this potential factor influencing FH and FHR binding. Therefore, and to further study the role of CR3, we used the U937 cell line and also an U937 cell line that overexpresses CR3 (CR3+ U937) (49). Compared to the wild-type U937 cells, CR3+ U937 cells bound more FH and FHR-1; however, FHR-5 binding was not increased (**Figure 3**). Because cell-bound C3-fragments may be derived from intracellularly synthesized C3, such as shown for U937 cells particularly upon activation (55, 56), we detected the presence of C3-fragments on the cells used in our assays. There was little to no C3-fragments detectable on the cell surface by flow cytometry in the case of both U937 cells and CR3+ U937 cells (**Supplementary Figure 1**), further supporting the role of CR3 in the binding of FH and FHR-1 in our previous assays (**Figures 1** and **2**).

### FH Family Members Modify PMA-Induced NET Formation

**Figure 4:**
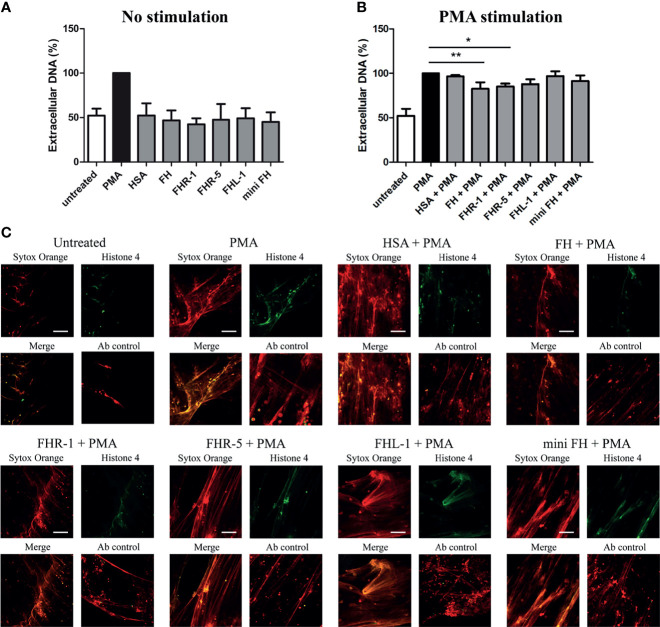
FH and FHR-1 decrease PMA-induced NET formation. 300 nM FH, FHR-1, FHR-5, FHL-1 or mini-FH were immobilized and incubated with neutrophil granulocytes in the absence **(A)** or presence **(B, C)** of 100 nM PMA for 3 h. As negative control, HSA-stimulated cells were used. **(A, B)** Cell-free NET was quantified by fluoroscan measurement using Sytox Orange DNA staining. PMA-stimulated samples were set as 100%. Data are means + SD derived from three different donors. Differences with *p* < 0.05 were considered statistically significant and compared to PMA treated samples (one-way ANOVA with Dunnett’s multiple comparison test, *p < 0.05, **p < 0.01). **(C)** Representative microscopic images of NET formation in the presence of PMA on 300 nM immobilized FH, FHR-1, FHR-5, FHL-1 and mini-FH using a 20× objective. NET formation was visualized by 5 µM Sytox Orange staining and histone H4 labeling. Scale bars represent 100 µm.

To investigate whether the binding of the studied FH family proteins to monocytes and neutrophils influence cellular functions, first we investigated how they influence NET formation of neutrophil granulocytes. Previously, we reported that FH alone does not influence NET production; however, after immobilization it significantly reduces the release of PMA induced extracellular DNA (16). Similar to FH, none of the FHRs alone had any effect on NET production (**Figure 4A**). PMA-induced DNA release, however, was slightly but significantly decreased on immobilized FHR-1, similar to FH (**Figures 4B, C**). FHR-5, FHL-1 and mini-FH however did not influence NET production even after PMA stimulation (**Figures 4B, C**).

### FH Family Members Influence Neutrophil and Monocyte Spreading

**Figure 5:**
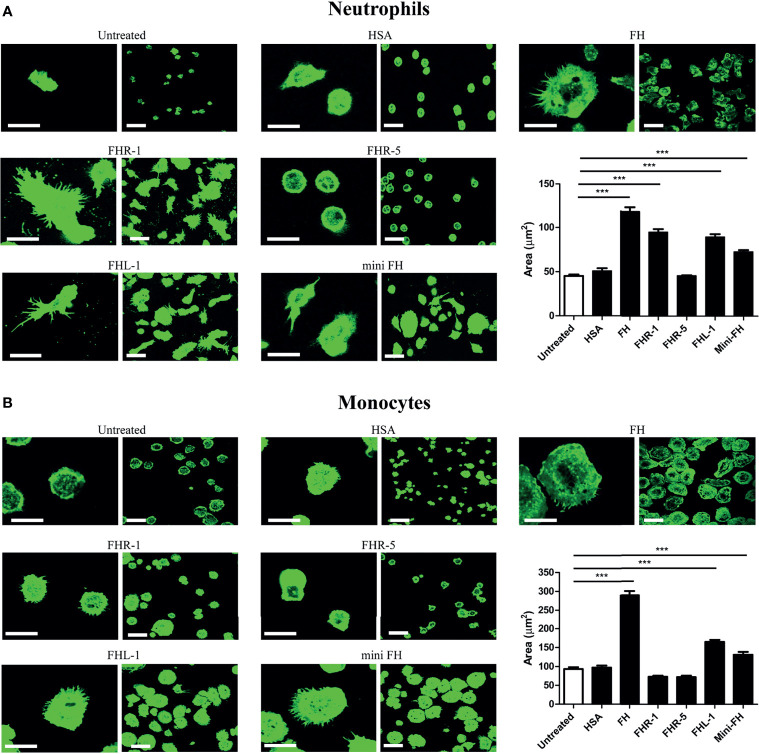
FH family proteins influence spreading of neutrophil granulocytes and monocytes. Confocal laser scanning images of **(A)** neutrophils and **(B)** monocytes in response to 300 nM of immobilized proteins on chambered microplate wells. The contact surface of the cells was monitored by labelling F-actin with phalloidin-A488. Original scale bars, 20 µm (left columns) and 40 µm (right columns). Confocal images shown are representative of four (neutrophils) or five (monocytes) independent experiments. The contact zone areas of phalloidin-A488 labelled neutrophils and monocytes were quantified using ImageJ software from 150 cells in each experiment. Error bars represent means + SEM. Differences with *p* < 0.05 were considered statistically significant and compared to untreated samples (non-coated microplate surface) (one-way ANOVA with Bonferroni’s multiple comparison test, ***p < 0.001).

It has previously been shown that FH, FHR-1 and FHL-1 serve as an adhesion ligand for neutrophils and that FH supports neutrophil spreading (14–17). Therefore, we investigated whether FHR-1, FHR-5, FHL-1 and mini-FH influence spreading of primary monocytes and neutrophils, an indispensable process of their extravasation and cell polarization (57). As shown in **Figure 5A**, neutrophil spreading was significantly increased on FH, FHR-1, FHL-1 and mini-FH coated surfaces compared to HSA, but FHR-5 had no effect. In the case of monocytes (**Figure 5B**), only FH, FHL-1 and mini-FH increased cell spreading significantly, whereas immobilized FHR-1 and FHR-5 did not alter the cellular morphology.

### FH Family Members Alter the Cytokine Profile of Resting Neutrophils and Monocytes

**Figure 6:**
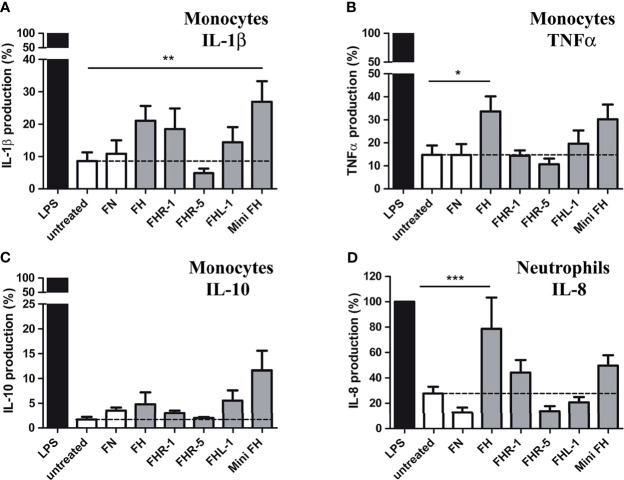
FH family members influence the cytokine profile of monocytes and neutrophil granulocytes. Cells were incubated in the presence or absence (untreated sample) of 300 nM immobilized FH, FHR-1, FHR-5, FHL-1 and mini-FH for 24 h, and IL-1β **(A)**, TNFα **(B)**, and IL-10 **(C)** secretion of monocytes and IL-8 secretion of neutrophils **(D)** were investigated by ELISA. As a positive control, cells were activated with LPS; 300 nM fibronectin (FN) was also used as an additional immobilized control protein unrelated to the FH family. Data represent mean + SEM of nine independent donors. Differences compared to untreated samples with *p* < 0.05 were considered statistically significant (one-way ANOVA with Bonferroni’s multiple comparison test, *p < 0.05, **p < 0.01, ***p < 0.001).

It has previously been shown that binding of FH to different surfaces influences the pro-inflammatory cytokine production of monocytes, macrophages and neutrophils (16, 27–29). To investigate whether FHR-1, FHR-5, FHL-1 and mini-FH are also able to modulate cytokine production of these cells, primary monocytes and neutrophils were incubated on surfaces where these proteins were immobilized, and cytokine secretion was analyzed after 24 hours. In the case of monocytes, only mini-FH enhanced IL-1β secretion significantly, however FH and FHR-1 also showed a tendency toward increased IL-1β production (**Figure 6A**). Additionally, FH and mini-FH caused increased TNFα release from monocytes, but only FH-treated samples reached statistical significance in the assay (**Figure 6B**). Interestingly, none of the proteins altered the anti-inflammatory IL-10 secretion significantly compared to the untreated sample (**Figure 6C**). In the case of neutrophils, immobilized FH increased IL-8 release, in agreement with our previous results (**Figure 6D**) (16). Similar to the case of IL-1β, the effect of FHR-1 and mini-FH on IL-8 production did not reach statistical significance, but followed a tendency to enhance the cytokine secretion.

### FH Family Members Modify LPS-Induced TNFα Response of Monocytes

**Figure 7:**
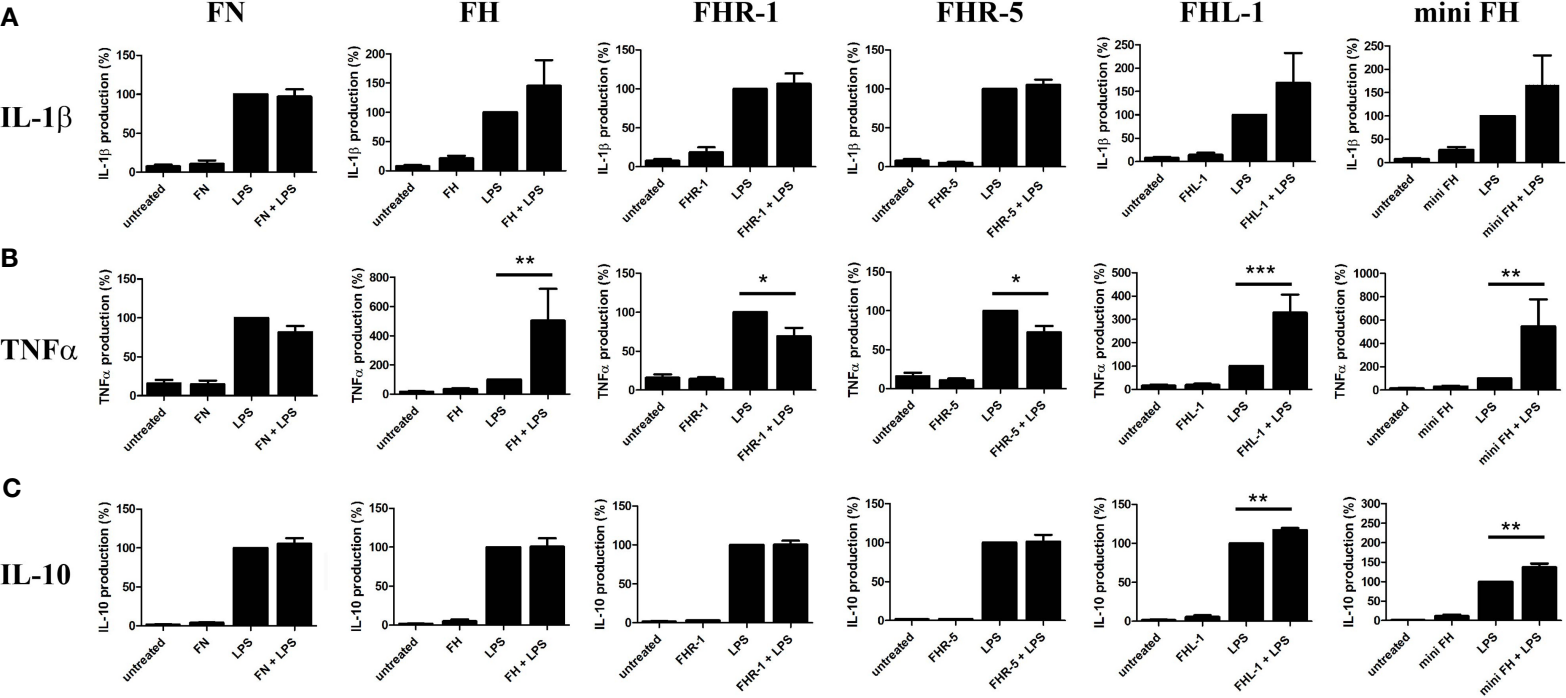
Combined effect of immobilized FH, FHR-1, FHR-5, FHL-1 and mini-FH with soluble LPS on cytokine production by monocytes. Human monocytes were stimulated with 10 ng/mL LPS in the presence or absence of 300 nM immobilized FH, FHR-1, FHR-5, FHL-1 and mini-FH for 24 h, and IL-1β **(A)**, TNFα **(B)**, and IL-10 **(C)** secretion were measured by ELISA. As a control, FN-coated cells were used. Data represent mean + SEM of experiments with five independent donors. Differences compared to LPS treated samples with *p* < 0.05 were considered statistically significant (one-way ANOVA with Bonferroni’s multiple comparison test, *p < 0.05, **p < 0.01, ***p < 0.001).

Since monocytes and neutrophils are able to bind FH family members and mini-FH, these proteins could influence the response of the cells when they are exposed to LPS from Gram-negative bacteria during infection. Therefore, we investigated the effect of FHR-1, FHR-5, FHL-1 and mini-FH on LPS-induced cytokine secretion of monocytes and neutrophils. None of the proteins affected IL-1β secretion compared to LPS alone (**Figure 7A**). However, when monocytes were incubated with immobilized FHR-1 or FHR-5 and simultaneously treated with LPS, a reduced secretion of TNFα compared to treatment with LPS alone was observed (**Figure 7B**). In contrast, FH, FHL-1 and mini-FH enhanced the amount of TNFα under the same conditions (**Figure 7B**). FHL-1 and mini-FH similarly enhanced IL-10 secretion compared to treatment with LPS alone (**Figure 7C**). In the case of neutrophilic granulocytes, none of the tested proteins influenced the LPS-induced, pro-inflammatory IL-8 release (**Supplementary Figure 2**).

## Discussion

**Figure 8:**
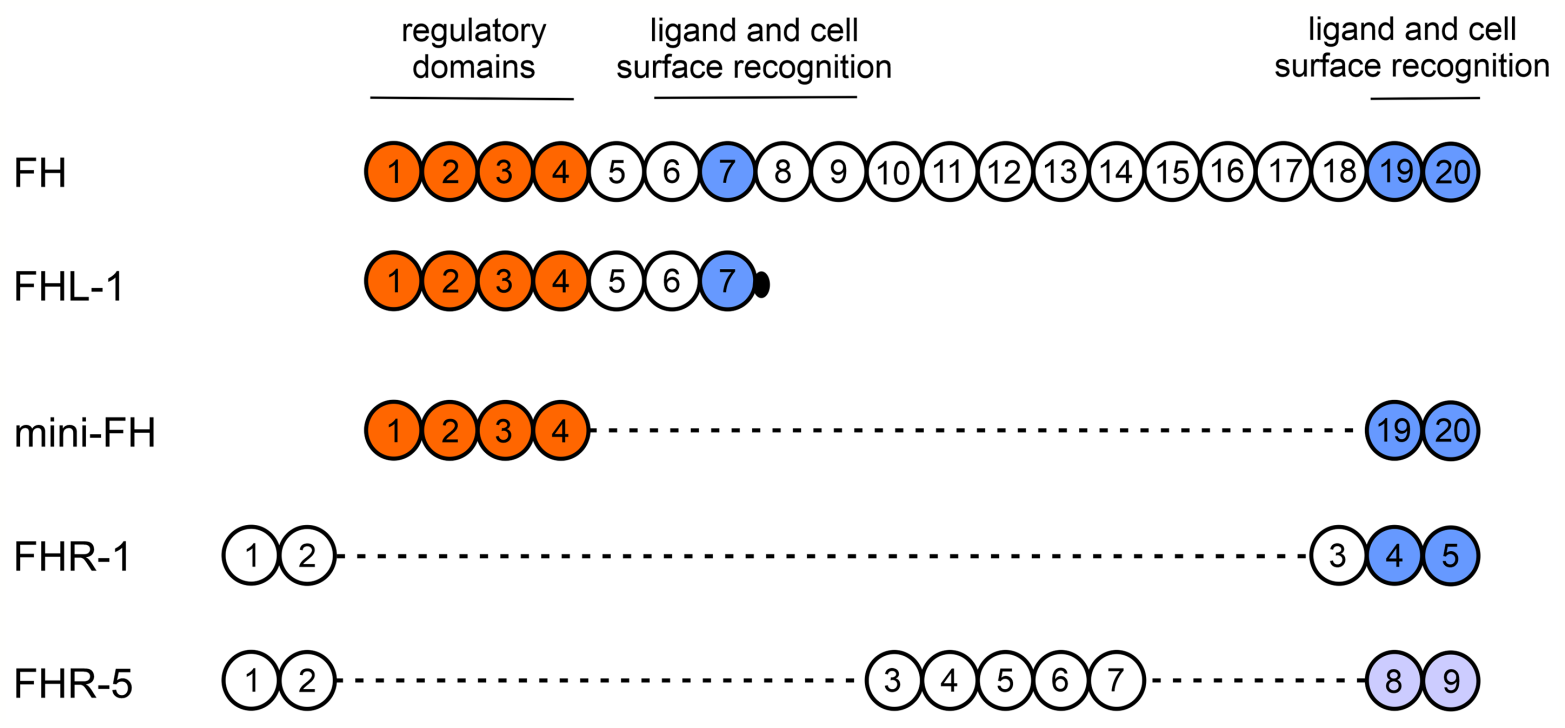
Schematic structures of FH, FHL-1, mini-FH, FHR-1 and FHR-5. The individual SCR domains are indicated with numbered circles and are vertically aligned based on homology among the domains of the proteins. Major functional sites of FH are indicated above. SCRs marked orange are responsible for the complement regulatory functions, such as cofactor and decay accelerating activities and also binding to C3b. FH shows 100% amino acid sequence identity with the corresponding SCRs of FHL-1 and mini-FH, but FHL-1 includes a unique four aminoacid long sequence at its C terminus. SCRs marked blue are involved in binding to several ligands and cell surfaces and also have a role in receptor binding. A previous study using various FH fragments and receptor-specific antibodies suggests that the FH C terminus mediates binding to CR3, whereas SCR7 to both CR3 and CR4 (15). Note that except for the C-terminal domains of FHR-1, FHR-1 and FHR-5 show lower aminoacid sequence similarity to the respective FH domains. The N-terminal domains of FHR-1 and FHR-5 mediate dimerization of these proteins.

Besides the canonical roles of FH family proteins in the modulation of complement activation, their non-canonical roles as modulators of cellular functions were also observed, but poorly examined yet (12, 33). Among blood cells, neutrophil granulocytes and monocytes were the most extensively studied in this regard as they are essential players in inflammation and innate immunity and they also influence the adaptive immune response. FH binding to neutrophils was first demonstrated in 1993 (58), and later it was shown to be mediated *via* CR3 (14–17) and to a lesser extent *via* CR4; the domains within FH responsible for this complement receptor binding were identified as SCR7 and SCR19-20 (15). These SCRs of FH show 100% amino acid sequence identity with the corresponding SCRs of FHL-1 (SCR7) or mini-FH (SCR19-20) (10, 33) (**Figure 8**). Therefore, FHL-1 and mini-FH have the potential to bind to the cell surface of monocytes and neutrophils similar to FH, which we confirmed by flow cytometry using recombinant proteins (**Figure 1**). FHR-1 binding to neutrophils was also shown before and is explained by its C-terminal domains that are highly similar to FH SCR19-20; FHR-1 binding could be inhibited by anti-CD11b and anti-CD18 specific mAbs but not by anti-CD11c (15). Moreover, binding of recombinant FHR-5, with less sequence similarity to FH, to the cells was also detected (**Figure 1**). These results are in agreement with our previous study where neutrophils were incubated with NHS and, in addition to FH, the binding of serum-derived FHL-1, FHR-1 and FHR-5 could be detected by western blot analysis using the cell lysates (15). The binding of FH to neutrophils could be inhibited by FHR-1 but not by FHR-5, further supporting the role of shared binding sites including CR3 for FH and FHR-1 on neutrophils (**Figure 2**). The relatively high amount of FHR-1 needed for significant inhibition of FH binding suggests that the binding of FH is stronger, e.g. because of the presence of at least two binding sites in FH *versus* a single site in FHR-1, and is also explained in part by the binding of FH to CR4, as well.

In addition to binding to neutrophil granulocytes, FH, FHR-1 and FHL-1 were described to influence certain cellular functions, but these proteins were studied only in certain aspects (14–17). Here, we studied all these proteins in the same assays and included FHR-5 as well as the artificial construct mini-FH (**Table 1**). The similar functional effect of mini-FH on the cells compared with FH can be explained by the shared SCR19-20 that are involved in FH binding to CR3 on neutrophils. FHR-5, despite of its strong binding capacity to both neutrophils and monocytes, had no effect on the investigated cell functions except for the TNFα response when applied in combination with LPS, clearly indicating marked functional differences among the otherwise related members of the FH protein family (**Figure 8**).

**Table 1:** Summary of the effects of FH, FHR-1, FHR-5, FHL-1 and mini-FH on different cell functions.

	FH	FHR-1	FHR-5	FHL-1	Mini-FH
**Neutrophils**					
Cell binding	+	+	+	+	+
NET release	–	–	–	–	–
PMA-induced NET release	↓	↓	–	–	–
Cell spreading	**↑**	**↑**	–	**↑**	**↑**
IL-8 production	**↑**	–	–	–	–
IL-8 production with LPS	–	–	–	–	–
**Monocytes**					
Cell binding	+	+	+	+	+
Cell spreading	**↑**	–	–	**↑**	**↑**
IL-1β production	–	–	–	–	**↑**
IL-1β production with LPS	–	–	–	–	–
TNFα production	**↑**	–	–	–	–
TNFα production with LPS	**↑**	↓	↓	**↑**	**↑**
IL-10 production	–	–	–	–	–
IL-10 production with LPS	–	–	–	**↑**	**↑**

(+ means binding; **↑** means significant increase and ↓ means significant decrease of each cell function; – means no effect).

Previously, we showed that neither soluble nor immobilized FH alone affected NET production. However, when NET formation was induced with PMA, immobilized but not soluble FH could significantly decrease the amount of extracellular DNA (16). In this study, FHR-1 but not the other FH family proteins or mini-FH had similar inhibitory effect on PMA-induced NET release as did FH (**Figure 4**). Prolonged or uncontrolled NET formation may enhance the development of inflammation or autoimmune diseases by providing autoantigens, such as DNA or histones. By inhibiting NET production, FH and FHR-1 may limit such undesirable reactions.

During inflammation caused by pathogens or other damage signals, extravasation of neutrophils and monocytes is essential. This is a multistep process including sequential rolling, firm adhesion/spreading and trans-endothelial migration (57, 59). Previous data suggested an adhesive function for FH in the case of neutrophils that is mediated *via* the complement receptor CR3 (14, 15). In addition, immobilized FH was shown to increase neutrophil spreading through the CR3 receptor, since in our previous study we found that specific antibodies against CD11b could inhibit this process (16), and to support the migration of both neutrophils and monocytes (15, 16, 24). Our present study confirmed the previously described increased neutrophil spreading on FH-covered surface (**Figure 5A**) and the same effect was observed on monocytes (**Figure 5B**). Immobilized FHL-1 and mini-FH had the same effect on both cell types (**Figure 5**). Interestingly, FHR-1 increased the spreading only in the case of neutrophils which suggests a different regulation process for the two cell types, and may be explained by the differential receptor expression (e.g., CR3 and CR4) on monocytes and neutrophils.

While all of the analyzed FH family proteins and mini-FH showed clear binding to monocytes and neutrophils, they also enhanced the attachment/spreading of the cells and influenced cytokine production, except for FHR-5. Since both cell types are key players in the initiation phase of the immune response, their cytokine production is essential during fighting against infections or can be an important factor in the pathogenesis of inflammatory diseases. We found significant increase in proinflammatory IL-1β secretion of monocytes cultured on mini-FH and slight increase when they were cultivated on FH, FHR-1 and FHL-1 coated wells. Similarly, TNF-α production by monocytes was significantly elevated on wells coated with FH and slightly on those with mini-FH. In the case of neutrophils, IL-8 production was monitored and immobilized FH was shown to enhance the secretion of IL-8 significantly, while FHR-1 and mini-FH induced only a slight increase (**Figure 6**). The direct role of FH family members in the cytokine production of different immune cells is not clear yet, since controversial findings have been described. Enhanced proinflammatory IL-8 and IL-1β production upon FH treatment was reported, however in most cases FH was applied in combination with other stimuli such as NET-derived DNA or *Candida albicans* (27, 28). Other studies support an anti-inflammatory role for FH, but again cells were treated with FH plus NET derived DNA, malondialdehyde acetaldehyde BSA or LPS and R848 as TLR ligands (29, 60, 61). We also tested the effect of the different FH family proteins and mini-FH on the cytokine production of LPS stimulated cells. In the case of TNF-α, FH, FHL-1 and mini-FH increased, whereas the FHR proteins FHR-1 and FHR-5 slightly decreased the level of this cytokine. In addition, FHL-1 and mini-FH slightly increased the secretion of IL-10. These results showing that the FH family proteins influence the LPS-triggered TNFα and IL-10 responses of monocytes may suggest their potential involvement in the regulation of inflammatory processes during infection. Interestingly, a recent study also found that mini-FH modulates complement-dependent IL-6 and IL-10 secretion by peripheral mononuclear cells in the presence of autologous serum; IL-10 release was increased by mini-FH when the cells were stimulated with LPS (62). When the effect of the FH family proteins was analyzed in isolated form, FH and FHR-1 treatment induced elevated IL-1β and IL-8 secretion (16, 26, 44) (**Figures 6** and **7**, **Supplementary Figure 2**). In our assays we applied immobilized FH family proteins, and in most other studies FH family proteins influenced cell functions only if they were immobilized on a surface of either a cell culture dish or yeast/bacteria particles. This supports the notion, that FH family members act as circulating molecular sentinels, that continuously monitor for altered structures or invading pathogens to which they can attach and then, in addition to modulating complement activation, they may bind to immune cells and modulate their function.

Soluble FH was found to be a direct modulator of monocyte differentiation since it can induce the differentiation of CD14^+^ human monocytes into immunosuppressive macrophages; this FH effect was inhibited in the presence of autologous plasma, suggesting that this function likely occurs only in tissues (63). Moreover, soluble FH can also generate an anti-inflammatory and tolerogenic state in monocyte-derived dendritic cells (61). However, in these experiments applying several days of co-incubation with FH, the possibility that the added FH may eventually coats the plate surface and thus also acts in an immobilized form, cannot be ruled out. Integrin receptors can sense differences between immobilized and soluble ligands (64, 65), which may explain why we observed an elevated, although not significant IL-1β production upon LPS stimulation on FH coat, while Smolag et al. found a significantly decreased production upon LPS stimulation in FH-treated monocytes (63).

In our experiments we used purified proteins to compare differences in direct cellular functions without adding any other complement component or serum, but it has to be noted that *in vivo* their relative local concentration and surface bound forms could influence the surface and receptor binding capacity of FH and FHRs, and thus cellular responses. Several questions remain to be answered regarding the non-canonical role of FH family members. The complement receptor CR3 (CD11b/CD18) and to a lesser extent CR4 (CD11c/CD18) were identified as the receptors for FH binding on immune cells; however, other candidates are also found, and the receptors for FHR proteins have not been clearly identified yet (14–16, 27, 58, 66). The similarity of FHR-1 domains 3-5 to FH domains 18-20 implies that the C-terminal SCR18-20 (or 19-20) domains are responsible for the binding and functional effect of FH and FHR-1 on immune cells. The identity of FHL-1 with FH SCR1-7 suggests, however, that SCR6-7 might play an important role as well in influencing immune cell functions. FHR-5 shows a lower degree of homology to both parts of FH, that may be the reason why we could not detect any direct functional effect of this molecule despite its prominent binding to the cells. These findings also suggest that FHR proteins could bind to other receptors than does FH.

While CR3 was identified as a FH receptor in the absence of deposited C3b (58) on human neutrophils and also on U937 monocytic cells (**Figure 3**, **Supplementary Figure 1**), the interaction of FH with cell surfaces is a complex phenomenon and is influenced among others by cell surface glycosaminoglycans and deposited other ligands, such as complement C3 fragments. The C-terminal domains of FH bind surface bound C3b *via* SCR19, while SCR20 binds sialic acids and glycosaminoglycans present on self surfaces which is important in the regulation of complement activation on the cell surface by FH (67–69); in addition, receptor-bound FH also retains its cofactor activity for C3b inactivation (16). The same C-terminal domains are involved in binding to neutrophils *via* complement receptors and mediating the non-canonical functions of FH (15). FH and FH SCR19-20 were also shown to prevent the adhesion of sickle cell disease erythrocytes to P-selectin and/or to CR3 by blocking C3b-cell interactions (70). Potentially, receptor-bound C3-fragments could also modify the interaction of FH and FHRs with cells. Moreover, it is unknown where exactly FH binds to its receptors. CR3 and CR4 are known to interact with several ligands, and their major ligand that promotes phagocytosis is iC3b on opsonized microbes and other particles (71). However, in our fibronectin and β-glucan induced NET model, iC3b used in combination with FH could not inhibit the effect of FH (16). Because there is evidence for ligand contact outside the I domain of CD18 (71) and the incubation of neutrophils with FH did not activate CR3 (72), it is likely that FH and iC3b bind differently. Further studies are warranted to better understand these interactions and their *in vivo* relevance.

In summary, we identify variable and context-dependent effects of FH family proteins on the function of human monocytes and neutrophil granulocytes. Furthermore, we show that FH-based complement inhibitors such as mini-FH, in addition to their effect on complement activation, may potentially also affect inflammatory cells. Further studies are needed to identify the exact receptors and mechanisms by which these proteins exert their functional effects on immune cells. A detailed characterization of the non-canonical functions of FH family proteins could provide a better understanding of their role in the clearance of different pathogens or host-derived debris and in the modulation of the resolution of inflammation and the pathogenesis of inflammatory disorders.

## Data Availability Statement

The raw data supporting the conclusions of this article will be made available by the authors, without undue reservation.

## Ethics Statement

The studies involving human participants were reviewed and approved by National Research Ethics Committee (TUKEB ETT, permission number 838/PI/12). The patients/participants provided their written informed consent to participate in this study.

## Author Contributions 

ÉK, MK, NS and MJ designed the experiments. ÉK, MK, NS, DH and AS performed experiments. MJ supervised the study. All authors discussed the data, revised and approved the manuscript. ÉK, MK, NS, AS and MJ wrote the manuscript. All authors contributed to the article and approved the submitted version.

## Funding

This work was financially supported in part by the Hungarian National Research, Development and Innovation Office (OTKA grants K 109055 and K 125219, and VEKOP-2.3.3-15-2017-00021), the Hungarian Academy of Sciences (grants nr. LP2012-43 and 0106307), the Institutional Excellence Program to ELTE (NKFIH-1157/8/2019, D11206), the European Union’s Horizon 2020 research and innovation programme under grant agreement No. 899163 (SciFiMed), and by the Kidneeds Foundation (Iowa, US).

## Conflict of Interest

The authors declare that the research was conducted in the absence of any commercial or financial relationships that could be construed as a potential conflict of interest.

## Publisher’s Note

All claims expressed in this article are solely those of the authors and do not necessarily represent those of their affiliated organizations, or those of the publisher, the editors and the reviewers. Any product that may be evaluated in this article, or claim that may be made by its manufacturer, is not guaranteed or endorsed by the publisher.

## Abbreviations

BSA, bovine serum albumin; FH, factor H; FHL-1, factor H-like protein 1; FHR, factor H-related protein; HSA, human serum albumin; FN, fibronectin; NET, neutrophil extracellular trap; SCR, short consensus repeat.
