# Supplementary material for: Complement Factor H Family Proteins Modulate Monocyte and Neutrophil Granulocyte Functions
Source: Front Immunol. 2021 Oct 4;12:660852. doi: 10.3389/fimmu.2021.660852 (PMC8521052; doi:10.3389/fimmu.2021.660852)
Supplement: Supplementary file 1 [file DataSheet_1.pdf]

Supplementary Material

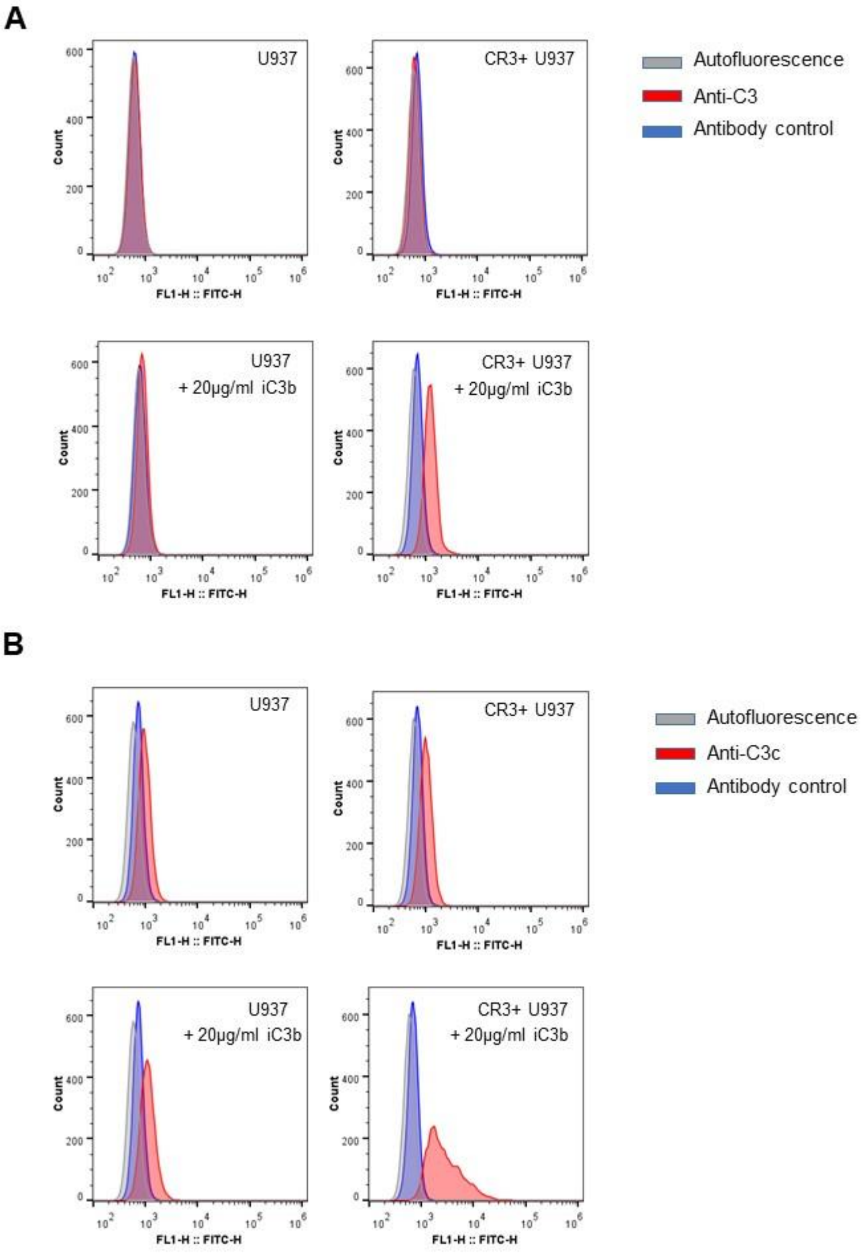

Supplementary Figure 1. Detection of C3-fragments on the surface of U937 and U937 CR3+ cells. The cultured cells were stained for the presence of cell-derived C3-fragments using (A) polyclonal anti-C3 and (B) polyclonal anti-C3c antibodies (upper panels). As a positive control, cells were preincubated with 20 µg/ml iC3b (lower panels). Representative histograms are shown.

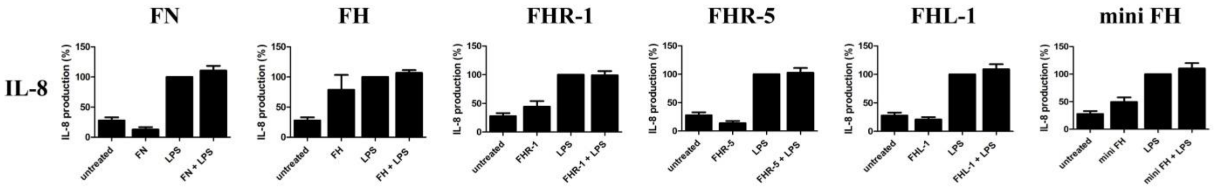

Supplementary Figure 2. Combined effect of immobilized FH, FHR-1, FHR-5, FHL-1, mini FH and soluble LPS on neutrophil IL-8 production. Neutrophils were stimulated with 10 ng/mL LPS in the presence or absence of 300 nM immobilized FH, FHR-1, FHR-5, FHL-1 and mini-FH for 24 h, and IL-8 secretion was measured by ELISA. As a coating control, fibronectin (FN) was used, for cytokine production negative and positive controls, untreated and LPS stimulated cells were used. Data represent mean + SEM of experiments with five independent donors. Differences compared to LPS treated samples with $p<0.05$ were considered statistically significant (one-way ANOVA, with Bonferroni's multiple comparison test).
